# The Importance of Being Relevant

**DOI:** 10.3389/fpsyg.2012.00309

**Published:** 2012-08-30

**Authors:** Snehlata Jaswal

**Affiliations:** ^1^Cognitive Science, Department of Psychology, Indian Institute of Technology RoparRopar, India

**Keywords:** feature binding, top-down and bottom-up processing, inhibition

## Abstract

This review aims at an understanding of the binding process by synthesizing the extant perspectives regarding binding. It begins with a consideration of the biological explanations of binding, viz., conjunctive coding, synchrony, and reentrant mechanisms. Thereafter binding is reviewed as a psychological process guided by top-down signals. The stages and types of binding proposed by various researchers are discussed in this section. The next section introduces Working Memory (WM) as the executive directing the top-down signals. After that it is described how WM works by selecting relevant sensory input, followed by a detailed consideration of the debate regarding objects vs. features with the conclusion that relevance is the key factor determining what is processed. The next section considers other factors affecting the selection of relevant input. Then, we shift focus to describe what happens to irrelevant input – whether it is discarded at the outset or is gradually inhibited, and whether inhibition is a perceptual or post-perceptual process. The concluding section describes the process of binding as currently understood on the basis of the literature included in the review. To summarize, it appears that initially the “object” is conceptualized as an instantaneous bundle of all features. However, only relevant features of stimuli are gradually integrated to form a stable representation of the object. Concomitantly, irrelevant features are removed from the object representations. Empirical evidence suggests that the inhibition of irrelevant features occurs over time and is presumably a process within WM.

Binding is the process whereby separate entities are linked together to form a unified, coherent representation of the world around us. Feature binding refers to linking various characteristics of the stimulus to form a coherent representation of the stimulus. Cognitive scientists postulate binding to be one of the basic processes in information processing ranging from object identification to consciousness (Crick and Koch, 1990, but, see Singer and Gray, 1995; Crick and Koch, 2003; Zimmer et al., 2006).

People from myriad backgrounds study binding. Philosophy debates whether the solution will be neurobiological, functional, computational, or a completely different kind. Empirical science focuses on how a person solves the binding problem at the neural or behavioral level. Rapid strides have been made in our understanding of the binding problem from the biological as well as the psychological perspectives, since the concept came into focus in the early 1990s. Nevertheless, researchers from diverse backgrounds often work within their microcosms, scarcely appreciating the similar nature of work in other researchers’ worlds. Moreover the diversity of current views regarding the process causes concern that the field may become excessively fragmented. Thus, this review is primarily attempting a synthesis of the existing views regarding binding. It particularly tries to bring together insights from the biological and psychological perspectives regarding binding. The theme that emerges is that relevance of features is crucial in the binding process. Only relevant features are integrated to form strong clear objects, whilst the irrelevant features are excluded. The review begins with a consideration of the biological underpinnings of the binding process.

## Brain mechanisms underlying feature binding: Conjunctive coding versus synchrony

The reality that is perceived is contingent upon information of diverse kinds located in many different areas of the brain. The binding problem exists because information about the features of every object in the external world is processed to disparate areas of the brain. Researchers have attempted to study different mechanisms whereby the brain brings together information that is initially represented in distinct areas of the brain. The modularity of the brain for processing different kinds of information is long established. Usually, however, we transcend these disparities, and accurately and effortlessly bind the myriad of information to create a holistic representation. So what is the underlying brain process, which binds together information that is represented in distinct areas of the brain? Almost all researchers assume that the answer lies in the identification of specialized neurons or networks that participate in the same cognitive process at the same time.

When Nobel prize winners Hubel and Wiesel provided evidence for conjunctively coding cells in the striate cortex of cats (Hubel and Wiesel, 1959, 1962), and monkeys (Hubel and Wiesel, 1968), it seemed clear that specialized neurons existed to code the different objects that are encountered in the environment. Researchers soon proposed that specialized cells attuned to specific conjunctions of features are responsible for binding, and that these cells come together in a workspace that enables the flexibility of binding and unbinding, and further processing. Fodor and Pylyshyn (1988) distinguished between vertical “modular faculties” and a distinct “central horizontal system” capable of sharing information across modules. Baars (1988) distinguished between a vast array of unconscious, specialized, parallel processors, and a single, limited capacity, serial workspace that allows exchange of information. Dehaene and Changeux (2004) proposed the “neuronal workspace hypothesis,” which distinguishes two computational spaces in the brain, each characterized by a distinct pattern of connectivity. They proposed a network of “specialized processors,” attuned to particular type of information, but sharing the characteristics of specialization, automaticity, and fast feed-forward processing, as well as “cortical workspace neurons” that break the modularity of the cortex because they are able to send and receive projections to many distant areas through long range excitatory neurons. However, the idea of binding due to specialized neurons had a problem with sheer numbers. The quandary was how to grapple with the numerous stimuli, account for transience of binding, and at the same time limit the huge number of conjunctively coding neurons required for all the binding operations. Computational models attempted to show how the magnitude of numbers could be significantly reduced. Mel and Fiser (2000) use an algorithm that gradually “acquires” a representation by choosing features that are statistically most likely to distinguish objects from their noisy environments. In the process, the system invokes any available strategy to limit processing to one or a small number of objects at a time, including biological mechanisms such as the fovea, or psychological ones such as attention. Proposing different types of bindings, O’Reilly and his associates suggest that higher order bindings can result from coarse coded representations. In fact, Cer and O’Reilly (2006) held that the posterior cortex deals with low order conjunctions with distributed coarse coded representations, the hippocampus deals with higher order conjunctions such as episodes or locations, whilst the prefrontal cortex actively maintains transient bindings in service of current goals. As is evident, these models reduce the problem of huge numbers only by proposing additions to the architecture or subdivisions within their models, either of which increase the complexity of the explanation.

Synchrony thus gained in popularity as an apparently more parsimonious alternative physiological explanation for binding. It was Von der Malsburg (1981) who first contended that a complex environment requires parallel processing of information related to different objects or events, and posited neural synchrony as the mechanism whereby such information is bound together. Singer and Gray (1995) suggested that binding is explained by transient and precise synchronization of neuronal discharges, discovered in their laboratory by Gray et al. (1992) in the cat striate cortex. Indeed, synchronization was later reported in species ranging from locusts (MacLeod et al., 1998), to cats and monkeys (Gray, 1999), and of course, in humans (Singer, 1999). The idea of synchrony assumes that binding occurs throughout the brain, synchronous firing of cortical neurons leading to binding of features. The proposal faces two problems. The first is with respect to how two (or more) bound objects are differentiated. Although oscillation between out of phase firing has been proposed as a possible mechanism to encode separate objects, it is difficult to imagine how such a precise timing mechanism is implemented, considering that there are always multiple objects in the external world, and in addition to that, the brain itself is a highly noisy environment. The second problem is related to the implication that the same neurons encode all binding operations, entailing that binding is transient. Precisely, the problem is how to account for permanence of representations after the stimulus is no longer there. Thus, synchrony seems to be an adequate explanation of binding, only for a single object, and that too only when it is present as a percept.

Nevertheless, physiological evidence exists for specialized processors as well as synchrony, and is hard to refute. Thus, many researchers tried to resolve the dispute between synchrony and specialized neurons by proposing different kinds of bindings, but in the process merely ended up reiterating the debate. Crick and Koch (1990) differentiated three kinds of bindings. First, bindings “hardwired” by genes or the experience of distant ancestors that determine the response to natural stimuli. Second, bindings learnt due to experience such as those required for recognizing familiar faces, or the letters of the alphabet; and third, transient bindings of novel stimuli which require focal attention. These are presumably based on neural synchrony, and if the stimulus is repeated often enough, develop into the second kind of bindings. Baddeley (2007) mentioned two types of bindings, passive binding, contingent on automatic processes; and active binding, which requires attention. The examples used by him suggest that while the former refers to binding elements of the natural world for which humans are “prepared” in an evolutionary sense, the latter type refers to binding of arbitrary, learnt elements. He further adds that long term episodic memory provides a third source of binding. Clearly these ideas are similar to the tripartite distinction by Crick and Koch (1990). VanRullen (2009) also distinguishes between hardwired binding of natural/frequently encountered objects, and on-demand binding of meaningless/arbitrary feature conjunctions, asserting that while the latter always requires attention, the former requires attention only if there is competition between multiple objects, thus emphasizing the inhibitory function of attention. Hommel and Colzato (2009) similarly hold that binding can take place through neural synchronization of all features present at a time, or because a stored detector exists for real/familiar objects. They too, admit the possibility of both processes acting together.

## Beyond perceptual integration: Recurrent processes

Despite this measure of acceptance, there is also a sense that mere perceptual integration of features, whether by synchrony or by specialized neurons, simply does not encapsulate all characteristics of bindings as coherent objects essential for all information processing. An object cannot be defined only as a cluster of features. Different features not only need to be integrated into a whole, but this “whole” needs to be consistent, distinct, and meaningful. Consistency would come from object persistence, distinctness is a concomitant of clarity and contrast from other objects; and meaningfulness implies that the object is of some consequence in the information processing sequence. To achieve these characteristics, the basic information supplied by separate features has to be transformed. This transformation is achieved by top-down processes that presumably select only the relevant features for processing.

At the physiological level, the selection of relevant features is done by the reentrant processes in the brain. These are the downward and lateral connections that feedback information to lower levels in the brain. As in any good communication system, the brain too relies on feedback mechanisms. In the visual system, for example, the lower level neurons in Area V1 send signals for forward processing, but it is also true that all higher visual centers have reentrant (downward) connections with Area V1. An important characteristic of reentrant connections is that they not only feedback to the original neurons, but also “receive” signals back from them. Communication between brain areas is therefore a continuous, iterative process.

The dichotomy between synchrony and specialized neurons is thus currently transcended by proposals that ascribe paramount importance to the evidence of reentrant processes in the brain. These top-down processes are linked to higher cognitive functions. Edelman (1978) first proposed that reentrant signaling may be important in integrating disparate cortical areas and higher brain functions. Damasio (1989) specifically argued that recall and recognition involve reactivation of the same areas that were involved in initial registration of conjunctions. This is done by means of “convergence zones” that enable retroactivation of multiple regions in the brain.

The crux of the reentrant theory is that brain processes are inherently iterative because of the hierarchical nature of the system and the fact that as information is processed in the higher areas the receptive fields become larger and lose their feature specificity. Thus one or more cycles are required to establish a stable representation. Reentrant processes confirm the correct features, resolve competition, and thus allow accurate bindings to take place (Di Lollo et al., 2000; Lamme and Roelfsema, 2000; Bullier, 2001; Hochstein and Ahissar, 2002; Hamker, 2003).

As far as the visual system is concerned, such signals emanate from the parietal cortex. Saalmann et al. (2007) studied how parietal output influences early sensory areas in macaques performing a visual matching task. They found that output from parietal areas increased activity in the early areas, and concluded that this represented top-down feedback from the parietal cortex to early sensory areas that helped to focus attention on relevant locations. Silvanto et al. (2009) used triple pulses of TMS over PPC to find that they led to excitation in the visual areas when applied unilaterally, demonstrating the top-down modulation of the visual areas by PPC.

Reentrant connections in the brain may also be combined with dynamic changes in synchronous activity to explain how the bound object is distinguished from the background, or indeed from other objects (Seth et al., 2004; Van der Togt et al., 2006). Thus reentrant processes are now accepted to be crucial for binding. Indeed, so compelling is this evidence that it has led to a rethink regarding the very concept of binding among many researchers. From the initial idea that all features are inevitably, instantaneously, and automatically bound together, there is a change to the view that reentrant connections confirm and integrate only some of the features in an iterative, resource-demanding process. There is a clear and discernible shift from the assessment of binding as a product to conceptualizing it as a process.

## Binding as a process guided by top-down signals

Treisman (1996) proposed three sequential mechanisms to solve the binding problem: selection of particular locations by a spatial attention window, inhibition of locations from feature maps containing unwanted features, and top-down activation of the location containing the currently attended object for further processing. She also speculated that reentry to area V1 or V2 mediated all these three different mechanisms, proposing that reentrant connections from parietal areas mediate spatial attention, from extra-striate areas mediate feature based selection, and from the inferior temporal cortex mediate object based selection. Treisman (2006) holds that the initial response of the brain is to activate feature detectors in the early striate and extra-striate areas that automatically connect to compatible temporal lobe object nodes, and perhaps inhibit the conflicting ones. The parietal cortex then controls a serial reentry scan of the V1 and V2 areas to retrieve the features present in each, and then these are combined to form integrated object representations or bindings.

Humphreys (2001) and his co-workers also propose a two stage account of binding (Humphreys et al., 2000, 2009; Cinel and Humphreys, 2006; Braet and Humphreys, 2009). The initial evidence for this two stage process came from a patient GK with bilateral parietal lesions (Humphreys et al., 2000). The patient could bind form elements into shapes, but could not integrate shapes with color. This prompted the idea that the initial stage of binding results in shapes, and thereafter, surface features are associated with the shapes. Cinel and Humphreys (2006) proposed that in the initial noisy stage, visual elements are weakly bound, and these bindings can dissipate unless they are consolidated into more stable and stronger representations by being reinforced by top-down attentional feedback modulated by the posterior parietal cortex. Humphreys et al. (2009) showed that form conjunctions were easier to detect than difficult feature targets by controls and parietal patients alike, whereas parietal patients were significantly impaired in detecting other cross domain conjunctions. Braet and Humphreys (2009) respectively used feature detection errors and conjunction errors as inversely related measures of feature detection and binding, and found that a patient with bilateral parietal lesions generated illusory conjunctions with unusually long display durations. Also, when transcranial magnetic stimulation was applied to the parietal cortex in normal participants, it led to an increase in conjunction errors, but only 150–200 ms after stimulus onset. Thus, they held that binding occurs due to reentrant communication emanating from the posterior parietal cortex.

Roelfsema (2006) postulates two mechanisms in the visual system responsible for binding – base grouping and incremental grouping. Base groupings are coded by single neurons tuned to multiple features, and reflect the selectivity of feed-forward connections. But, all possible feature combinations cannot be coded by dedicated neurons. Therefore, a second, flexible form of grouping called incremental grouping needs to be posited. Incremental grouping augments the responses of the set of neurons coding separate features that are bound in perception. It takes more time than base grouping because it relies on horizontal and feedback connections, besides the feed-forward ones. The modulation of neuronal response strength, i.e., the firing rate of neurons, during the incremental grouping stage parallels the behavioral fact that attention is directed to features that are indicated by the enhanced neuronal response, and those features are then bound together. Base grouping takes place initially in the system, followed by incremental grouping in the cortex. This basic theory has been enhanced to propose a computational model that predicts figure-ground separation as well as binding (Jehee et al., 2007).

Zimmer et al. (2006) distinguish between transient binding and more durable binding, implying that different mechanisms probably bind features at different points in time, and/or there is a process of consolidation that transforms transient binding into durable binding. Shifting the focus to a very late stage in the binding process and thus proposing an integrated model of binding in WM and long term memory, Murre et al. (2006) also distinguish between transient and permanent binding, suggesting that while the former reflect the capacity of WM to select task relevant information for processing, the latter is the capacity of the neural system to store coherent patterns in LTM. Their emphasis, nevertheless, is that there is a constant interaction between these two. “What is transiently bound in WM governs what is temporarily and eventually permanently bound in long term memory. In turn, what is permanently bound affects transient binding in WM. The interplay of these binding processes determines how the brain develops into a structured system that is cumulatively correlated with its environment, thus implementing a process that is able to lift itself to higher levels of cognitive functioning” (Murre et al., 2006, p. 244).

Emphasizing the top-down factors even more, Hommel and Colzato (2009) propose that memory for a binding is controlled by two kinds of priming processes. Offline priming happens before the stimulus is presented, due to foreknowledge about the relevant features, task instructions, manipulation of mental set, etc. Online priming is induced by stimuli that have entries in long term memory, such as familiar objects. These are detected in a non-selective fast feed-forward sweep, followed by recurrent processes refining the input according to the operating principles of the attentional set for that task.

### Summary so far

In line with these ideas, one may conclude that binding is a continuous process that begins with the sensory input which goes to myriad areas of the brain and ends when the bound object emerges in memory such that it is strong enough to be manipulated further for higher cognitive processes. Features are initially perceived together either through synchrony or by neurons coded to detect conjunctions. This integration is a largely automatic, non-conscious process, which thereafter is refined by iterative processes and ultimately allows differentiation and dissemination of information in conscious states.

## Working memory as the executive directing top-down reentrant processes

The process of binding inevitably begins with the physical continua impinging on the sense organs. But it is equally true that the information regarding stimuli does not enter an empty box. The brain has its own ideas! The top-down control of behavior by mental representations of goals, instructions, and ideas is perhaps as undeniable as the source of behavior in bottom-up processing of stimulation. A logical assumption is that since the reentrant signals emanate from the cortical regions higher up in the processing hierarchy, they reflect top-down modulation of the process of binding. The question still remains as to what is at the “top”? What directs the reentrant processes? Working Memory (henceforth WM) seems to be the executive guiding all processes of the brain in the service of current goals. The prototype model of WM (Baddeley and Hitch, 1974; Baddeley and Logie, 1999) emphasized that different kinds of information are processed by distinct mental systems that act together to deal effectively with tasks confronting a healthy human adult. Baddeley and Hitch (1974) distinguished two subsystems, the phonological loop and visuo-spatial sketchpad, and a control system called the central executive. To deal with the fact that the information processed in these subsystems is often combined, and indeed, is at some stage also linked to information in the long term permanent store, Baddeley (2000) proposed a fourth component, the “episodic buffer.” The episodic buffer was initially theorized to be controlled by the executive, but was primarily a storage system linked to conscious awareness, that binds together information from different sources in episodes. Thus, the episodic buffer was proposed as the answer to the binding problem (Baddeley, 2003).

Logie (2003) conceptualized WM as a mental workspace that deals with integrated objects identifiable on the basis of prior knowledge. He maintains that sensory input reaches WM only after it has activated the knowledge base. Thus what reaches the workspace is the result of an amalgamation of the sensory input and the knowledge base. Since another source of input into the workspace is retrieval generated by processes in WM such as imagery, he holds that WM does not act as a gateway for processing information into LTM (substantiated by Van der Meulen et al., 2009). The workspace model implies that feature binding takes place concomitantly with or before the object representation evokes prior knowledge, which in turn, precedes the manipulation of the object in the mental workspace. Nevertheless, other kinds of binding, such as between objects and their semantic associates, or between percepts and images, or between images, or among sequentially presented objects, presumably takes place in the workspace that is WM. The basic units in WM are thus perceived objects. Nevertheless, the model can also be interpreted to accommodate the idea that features themselves might evoke prior representations in LTM and then the processes of WM refine the representation so that it emerges as an object.

Although there are many interpretations of WM as a concept and consequently many different models exist in current literature (reviewed by Miyake and Shah, 1999; Osaka et al., 2007), consensus exists on two important characteristics. First there is a general assumption that physiological level explanations are tenable for the WM phenomena observed at the behavioral level. Second, attention has a crucial and largely inhibitory role to play in all models of WM. Both these characteristics imply that WM is the top executive, the “controller,” managing the stimuli. It is in this sense that one may conceptualize WM as the source of top-down influences directing recurrent processes.

## How does working memory work – by selecting relevant input?

The advocates of top-down processes regularly invoke and use the concept of WM as the top executive in their theories. The biased competition theory by Desimone and Duncan (1995) proposed that WM content in terms of instructional set, task goals, etc., facilitates the selection of matching sensory input. The biased competition model rests on the assumption that attending to an object causes a bias signal to be sent by higher areas to the lower sensory areas which increases their tonic activity without necessarily increasing the neural responses evoked by the external stimulus itself. Behaviorally, this assumes that incoming sensory stimulation is matched with a template which specifies the relevance or otherwise of the received stimulation. Although Duncan (2006) concedes that in principle, competitive bias can begin anywhere in the system and then spread to the higher and/or lower levels, he also reiterates the role of task relevance and an associated pattern of fronto-parietal activity that he calls the multiple demand pattern because it is produced by many different kinds of cognitive demands. No wonder that his theory is usually taken to be a prime exemplar of the emphasis on top-down processing.

Based on studies using single unit recordings in macaques (Chelazzi et al., 1993), it was held that a state of competition always exists among the variety of sensory inputs at any moment. Stronger sensory inputs usually win out, but the representations in WM bias the competition such that inputs matching them are the ones that are strengthened and selected for further processing. The contention that competition is essential for attention to emerge is supported by neuroimaging evidence that the posterior parietal cortex, which is activated when visuo-spatial attention is focused, promotes feature binding when there is a potential for confusion with the simultaneous presence of other objects. Kastner et al. (1998) used fMRI evidence to substantiate that when stimuli are simultaneously presented, their cortical representations interact in a competitive and suppressive way in the ventral (object recognition) pathway. However, this was not evident when stimuli were presented sequentially. In a second experiment, spatial attention focused on the objects was found to counteract the suppressive effect, and more so in the simultaneous as compared to the sequential presentation condition. Using fMRI, Shafritz et al. (2002) established that the posterior parietal cortex was active when multiple objects were simultaneously presented, but not when they were sequentially presented in the same location (at fixation).

## What is selected as relevant input? objects or features?

Currently, there is conflicting evidence regarding the level to which distracters are represented in the brain. Some researchers propose that all objects and features are automatically and implicitly represented in the brain up to a level that excludes semantic processing (reviewed by Thoma et al., 2004). Nevertheless some studies indicate that even unattended objects are habitually processed to the semantic level (Pins et al., 2004; Altmann et al., 2005). Attempting a resolution, Martinovic et al. (2009) used EEG to find evidence that induced gamma band activity was enhanced due to the presence of distracter objects under low load conditions, thus providing evidence for cortical representation of distracters. However, as perceptual load increased, attentional selection played a more important role, and gamma band activity was limited to the attended object with a general suppression of all activity linked to surrounding information. This corroborates Duncan’s views regarding suppression of distracters by attention in consonance with top-down directions.

Emphasizing the integrated nature of processing of objects, Duncan (1996, 1998, 2006) held that since the object features are encoded in an integrated fashion across different cortical regions, if attention is directed to one feature, all features of the object, whether relevant or irrelevant, become dominant in their respective regions of the brain. Support for this idea came from fMRI data by O’Craven et al. (1999) who found that neural activity increased in response to the attended as well as non-attended task-irrelevant attribute of the stimulus. Nevertheless, their studies also provided evidence for differential level of activity, with the absolute amount of activity being stronger for relevant features than for irrelevant ones in the attended object. More definitive data were provided by Schoenfeld et al. (2003) who recorded event related potentials as well as event related magnetic fields together with fMRI to find that the irrelevant feature was activated rapidly enough to participate in the perceptual integration and binding of the attended object. Using event related potentials, Winkler et al. (2005) found evidence that pre-attentive binding of relevant as well as irrelevant features occurs “normally” in visual as well as auditory modality, and that attention is required for correct binding only under special circumstances when high load displays are processed under high time pressure.

Thus, in considering the difference between relevant and irrelevant, the distinction between features and objects is crucial in Duncan’s theory. Duncan (1980) asserted that only targets are selectively processed through the limited capacity system, non-target objects are identified and rejected by initial parallel and unconscious processes. Nevertheless, this selectivity is not assumed to operate on features. Duncan (1984) showed that perceptual identification of properties inherent in two different objects is much more difficult than when the features are combined in a single object. However, if two features are combined within a single object, the visual system finds it as easy to encode a combination of two features such as orientation and texture, as to encode them separately. Duncan (1998) provided evidence that it is also hard to identify two separate targets presented within the same modality, though there is no problem in detecting targets that differ between modalities. Thus, the features of an object are integrated such that they are processed together in an all or none fashion. Directing attention to a selected object enhances the representation of all its features together (Egly et al., 1994; Duncan et al., 1997). The objects compete with each other and the winner is processed further at the cost of widespread suppression of the distracters or the ‘to be ignored’ objects. Competition is biased and ultimately resolved in favor of task relevant objects, and typically this state is achieved over 100–200 ms and is sustained by attention.

Luck and his associates also contend that the basic units on which VWM operates are objects rather than features. Luck and Vogel (1997) held that VWM was object based because remembering one feature such as color allowed the recall of another without any additional cost. Vogel et al. (2001) confirmed that VWM can hold three to four chunks of information, be they features or bound objects. This evidence suggests that VWM stores integrated objects rather than features, and objects are thus the basic units of VWM. Woodman and Luck (2007) tested the prediction of the biased competition model that a match between the template held in WM and the sensory input always leads to a facilitation of performance. They used a dual-task paradigm and asked participants to perform a visual search task while maintaining object representations in VWM at the same time, but found no such facilitation of performance. Nevertheless, the reaction time was faster for matching distracters. When the participants knew beforehand that the target would never match the item retained in memory, they could direct attention away from the items that matched the WM representation. Thus they found an inhibitory effect and concluded that participants can use the content of WM strategically to inhibit as well as facilitate attentional processing. Moores and Maxwell (2008) also found that prior stimuli in WM captured attention even in the absence of bottom-up priming, and influenced the response of the participant, despite the influence being detrimental to the task. Indeed one important purpose of VWM is postulated to be the control of eye movements (denoting attention), specifically the initial direction and subsequent correction of gaze toward particular objects in visual search (Hollingworth et al., 2008; Hollingworth and Luck, 2009).

In contrast to these theories emphasizing the top-down nature of processing of integrated objects, are accounts of behavior that stress the role of different features of the stimuli to be processed. These accounts vary in their espousal of top-down mechanisms. For example, the feature integration theory (Treisman and Gelade, 1980; Treisman, 2006) and contingent capture theory (Folk et al., 1992, 1993; Folk and Remington, 2006) ascribe paramount importance to top-down factors implemented through attention. At the other extreme are the accounts of stimulus driven capture (Theeuwes, 1992, 2004), and dimension weighting (Muller and Krummenacher, 2006), which primarily emphasize the importance of bottom-up factors in capturing attention. Nevertheless, they are similar in stressing one or more features as being relatively more important in the process of binding.

The feature integration theory (Treisman and Gelade, 1980; Treisman, 1988, 1998) suggests that attention to particular locations is the most important factor in feature binding, implying that all features present at a particular location are inevitably bound together if attention is focused on them. Treisman and Zhang (2006) reiterated the importance of locations in binding in VWM as well. This view makes binding a relatively automatic process triggered off by attention to particular locations. Basically, it postulates a master map of locations, and as attention is focused on any area of this map, the object in that location is encoded. Also, while detection of features is contingent on independent maps for each feature, other types of searches, particularly conjunction search, is driven by the master map of locations that integrates information from other maps to produce the signals that make each stimulus salient (Treisman and Sato, 1990; Wheeler and Treisman, 2002).

Kahneman et al. (1992) proposed the object file theory, according to which objects are primarily identified by their positional marker or spatial index. Thereafter, other properties of the object, color, shape, etc., are associated with the spatial index. Spatiotemporal continuity is essential for maintaining object file representations, whereas non-spatial properties such as color and shape are unimportant. Direct evidence in support of this idea comes from the object reviewing paradigm (Kahneman et al., 1992; Mitroff and Alvarez, 2007), the multiple object tracking paradigm (Pylyshyn, 2004), visual search in dynamic displays (Horowitz and Wolfe, 1998; Alvarez et al., 2007); and developmental evidence showing that young infants rely on spatiotemporal rather than surface features or identity information to make sense of their visual world (Feigenson and Carey, 2005).

Applying the feature integration theory and the idea of object files specifically to the binding process, Treisman (2006) maintained that pre-attentively, features, and locations are registered in different maps, and focused attention binds them together. She mentioned three components of the binding process, and suggests that we shift attention in space to select one object after another, suppress features of other objects, and finally bind selected features together into “object files.” Note that she contends that initially, features are processed in parallel and stored as separate traces, which are only inhibited, but not completely eradicated, in the binding process. Revisiting the feature integration theory, Chan and Hayward (2009) have provided fresh evidence for dissociation between feature detection and localization, involving respectively parallel and focal search.

To completely grasp the implications of Treisman’s ideas, it is instructive to contrast them with Duncan’s model. One difference is their view of binding. For Duncan, binding happens at a very early stage in the visual process and the basic units in his theory are bound representations or objects. For Treisman, binding is a process of continuous refinement, during which features become linked to a master map of locations. Features remain bound only as long as attention is focused on them. Any irrelevant features continue to exist in an attenuated form. Another related but important point of distinction lies in their disparate view of the role of attention in binding. While Treisman views attention to be a selective process essential for binding, Duncan assumes that features are already bound into objects (probably through conjunctive coding by neural detectors) and then biased competition between objects occurs accompanied by a state of attention. Attention is thus an emergent property of the system, and the mechanism that aids top-down biased selection of some objects over others. It follows that Treisman holds attention to be an earlier process than Duncan. Finally the most important influence in the process of attention for Treisman is location, so attention is basically spatial in nature, whereas for Duncan, it is an emergent property of the system that is weighted in many ways, but essentially by task relevance more than anything else. Despite these differences, both agree that attention is necessary for binding separate features into a coherent object.

Treisman’s insistence that attention was primarily spatial also conflicted with experiments showing attention capture by abrupt onsets, the tendency of anything unusual in the field to attract involuntary attention, even if participants are set to ignore them (Remington et al., 1992). Nevertheless, the contingent capture theory (Folk et al., 1992, 1993; Folk and Remington, 2006) holds that attentional capture, as for example, by abrupt onsets, is contingent on top-down attentional control settings. This was because the original experiments showed that abrupt onset cues captured attention when the task was to identify onset target, but color cues captured attention when the task was to respond to color targets. Folk et al. (2008) have established that non-spatial attention is also subject to capture that is contingent on top-down settings. In their experiments, a change in the color of the distracter such that it matched the target, decreased target detection. Folk et al. (2009), showed that even non-spatial distracters which did not capture attention, nonetheless, influenced responses to a target.

The guided search model (Wolfe et al., 1989; Wolfe, 1994) had a rather different concern regarding the feature integration theory. Wolfe et al. (1989) pointed out that except for locations, the feature integration theory did not differentiate between other features of the stimuli. Further, it presumed that parallel processing of features in the initial pre-attentive stage did not have any impact on the later attentive serial search. The guided search model proposed that the features which were processed in the parallel stage guided attention in the subsequent serial stage, primarily by dividing the stimuli into distracters and probable targets. Further, they provided evidence that search for conjunctions defined by three features was more efficient than conjunctions of two features, simply because more number of features guided search for triple conjunctions. Wolfe (1994) acknowledged the special role of location by modifying the model to suggest that the output of processing in the initial massively parallel stage guided spatial attention and thus the second serial stage processed input from a limited portion of the visual field. Note that this reverses the sequence of the relative influence of location and other features postulated by the feature integration theory which holds that other features are attached to a master map of locations and hence spatial attention precedes and guides attention to features (Treisman and Sato, 1990).

The idea that each feature is coded within its own feature map was extended by Vidal et al. (2005) to include the notion of “structural gist.” Their Experiments 1, 2, and 3 using a change detection task with a study-test interval of 1000 ms showed that it was more difficult to detect changes of only color, only shape, or only orientation, in a target item, if the distracters also changed on the same dimension as compared to a condition where there was no change in distracters. Experiment 4 showed that change detection was impaired when an item that remained on screen during the study-test interval changed in the same dimension as the target, demonstrating that encoding relational information was possible even when it was not presented simultaneously. In Experiments 5 and 6, they compared conditions when distracters could change on the same dimension as the target, or on a different dimension. Changes in a different dimension, however, did not have an effect on performance, whereas changes in the same dimension did affect performance. It was more difficult to detect feature changes when the distracters also changed features on the same dimension, as compared to when the distracters changed on some other feature dimension. Thus they proposed that each item in each feature map is encoded in terms of individual as well as configural information. The effects of relational information are particularly strong within each feature map. Their experiments considered only changes in colors, shapes, and orientation, keeping location constant. However, Jiang et al. (2000) had earlier reported that detection of changes in color was impervious to change in locations of non-targets.

The dimension weighting account (Muller and Krummenacher, 2006), which may also be considered an extension of the guided search model, holds that attentional weights are allocated to basic visual dimensions (such as color, orientation, etc.), on the basis of stimuli defined by features (red, tilted, etc). Enhanced feature contrast within a dimension, e.g., red vs. green rather than yellow vs. green, and amplified saliency signals about a dimension to the overall saliency map, can facilitate detection of targets defined by that dimension, or alternatively target detection may be delayed if the target dimension changes across trials, shifting the weight to a new target defining dimension. They propose that the dimension weight can never be set to zero and indeed, may reflect the speed of processing associated with various dimensions. Weighting effects are proposed to be pre-attentive, influencing dimension based saliency signals before the overall saliency computation which is the basis of attentional selection of objects. Nevertheless, weight shift can be modulated through expectancies set up by cues, instructions, past experience, etc. In this sense the role of top-down processes is acknowledged. Muller et al. (2009) used the singleton salient distracter paradigm and showed how distracter influence varied with relevant practice, such that participants could learn to suppress distracters depending on the incentive to use suppression which in turn was presumed to vary with the probability of occurrence of the distracter. Nevertheless the costs of dimensional cueing in these studies could be, in part at least, due to task switching in general. Pan et al. (2009) studied the effect of dimensional cueing when the relevant dimensions were known to the participants. In fact, participants were explicitly instructed to prepare the relevant dimension on congruent trials and discard the irrelevant dimension on incongruent trials. Participants received a dimensional cue to be held in memory, and were subsequently tested on it either before or after a test of attention. Response latency was more on incongruent trials and less on congruent trials as compared to neutral trials. The benefits of congruency were enhanced when the cued dimension had to be held in memory throughout the trial, i.e., when the memory test was given after the attention test. This study demonstrates that the contents of WM can have an effect, and in fact, have more positive than negative effects on performance.

Theeuwes and his colleagues have consistently adhered to a strict bottom-up account of behavior (Theeuwes, 1992, 2004; Theeuwes et al., 2006). Their paradigm essentially investigates the effect of a singleton distracter defined by a different dimension than the one defining the singleton target. Theeuwes (1992) used a distracter defined by color (the only red among all green), and a target defined by shape (the only diamond among circles). The initial check confirmed that RTs were quicker to color than to shape, showing it to be more salient. Then participants performed under two conditions, one in which the distracter was present, and the other in which it was not. Results showed significant distracter interference in that RTs were significantly slower when the distracter was present. The embarrassing question for adherents of top-down influences was why participants were unable to ignore the distracter; despite the fact that they knew what dimensions were defining the target and the distracters. Schreij et al. (2008) reported that abruptly occurring distracters produce costs in performance even in the presence of a top-down set for color. They argue that these results show that abrupt onset of new objects captures attention independent of a top-down set and thus, provides conclusive evidence against the idea that attention capture is contingent only on top-down attention control settings.

It is, of course, possible to take an eclectic view of the tripartite competition among researchers who have proposed objects, locations, or features to be the units of visual processing. Humphreys (1998) proposing a dual coding account of representation of objects in space, contends that we have a rather poor representation of space *per se*. However, objects are spatially represented in two ways, within object representations, where elements or features are encoded as part of objects, possibly in the ventral stream with some dorsal involvement; and between object representations, where objects are coded in relation to each other, presumably involving the dorsal stream. Both these kinds of representations exist in parallel. Visual processing capacity is limited by the competition to encode elements within an object, the number of objects that can be encoded at the same time, and the relevance of within object or between object representations to the task. In this view, unlike the feature integration theory, there is no attempt to assign a special role to locations. Indeed, the bottom line is that the objects in space are important. Space in itself is not significant. The feature that is important here is form, for form elements are bound in the absence of focal attention and are later associated with surface features such as color. In giving this account, Humphreys also diverges from Duncan, and proposes that competition may exist within the elements of an object as well, and further, this competition can be biased by task relevance. Thus the differential effect of features can itself be influenced by top-down factors.

Linnell and Humphreys (2004) have shown how object based selection can overrule the central bias, the fact that attention is primarily directed at fixation and performance decreases as eccentricity of the targets increases. Linnell and Humphreys (2007) used the odd man out paradigm of visual search and found that when the participants knew in advance about the feature defining a target, detection was enhanced due to grouping on that target feature, and the participants then limited search to that group only. This grouping by features overruled the central attentional bias by allowing the grouping of peripheral targets with centrally presented distracters. They concluded that visual search can be space, object, or feature based, and in fact, performance is often determined by an interaction of all three. The real winner is top-down modulation which directs which of these three are relevant to the task at hand.

## Other factors affecting the selection of “relevant” input

Current research has largely moved away from the debate among objects, locations, and features, to focus on *how* top-down WM factors influence the encoding and retention of stimuli. An influential idea delineating how WM deals with distractors is the load theory of selective attention and cognitive control (Lavie et al., 2004; Lavie and De Fockert, 2005, 2006). It suggests that WM provides goal-directed control of visual selective attention by decreasing interference by goal-irrelevant distracters. Lavie and De Fockert (2005) tested this idea with the singleton paradigm. They showed that attention capture by an irrelevant color singleton during shape search critically depends on availability of WM to the search task. When WM was loaded by a concurrent yet unrelated verbal short-term memory task, capture increased. Increasing WM load also results in greater distracter interference in Stroop-like tasks (De Fockert et al., 2001; Lavie et al., 2004). In fact, increasing WM load leads to greater distracter interference whereas increasing perceptual load reduces distracter interference (Lavie et al., 2004). Park et al. (2007) demonstrated that the type of WM load is crucial to this effect using the flankers task with houses and faces as stimuli. Distracter interference increased when the memory load items overlapped with the targets, but decreased when they were similar to the distracters. These findings suggest two selective attention mechanisms: a perceptual selection mechanism serving to reduce distracter perception in situations of high perceptual load that exhaust perceptual capacity in processing relevant stimuli and a cognitive control mechanism that reduces interference from perceived distracters as long as cognitive control functions are available to maintain current priorities (low cognitive load).

Forster and Lavie (2008) reasoned that in real life situations, there is as much need to avoid external irrelevant distracters as there is to suppress relevant distracters. Laboratory studies usually focus on relevant distracters alone. Thus they compared the effects of perceptual load on task-irrelevant and task relevant (response competing) distracters. They found that an entirely irrelevant distracter can interfere with task performance to the same extent as a response competing distracter. High perceptual load in the task eliminated the effect of both types of distracters with similar effectiveness. Forster and Lavie (2007) showed that although individual differences in reported distractibility were correlated with distractibility in a response competition task performed in the laboratory, high levels of perceptual load in the task reduced distracter interference for all participants. Forster and Lavie (2009) demonstrated how a high perceptual load, demanding task relevant processing, concomitantly decreased the frequency of task unrelated thoughts, and thus reduced “mind wandering.” When one needs to focus on a task, it is easier to inhibit both external and internal sources of interference.

Olivers et al. (2006) found that singleton distracters interfered more with visual search when an additional memory task had to be performed at the same time. The interference effect was even stronger when the distracters were virtually the same or related to the object held in memory. Houtkamp and Roelfsema (2006) studied whether items in WM influence the deployment of attention. Using line drawings of simple objects, they asked participants to remember two objects. After a blank interval of 1000 ms, while the participant was instructed to search for one of the two items as a target, the other memory item was sometimes presented as one of the distracters in an array of items. They found that the distracter had an effect only if the target was absent. Whenever, the target was present, the memory item had no effect as a distracter. Eyes were unlikely to be fixated on the distracter, and if they did, fixation duration was very short. Thus attention was primarily oriented toward the target, and memory items had an effect only if the target was absent. The special processing of the target has been found with objects in real world scenes as well. Details of targets and distracters related to targets are better retained than the distracters that are unrelated to the targets, maybe because they are looked at more frequently as shown by eye movement recordings (Williams et al., 2005). Brisson et al. (2009) investigated whether contingent capture required capacity-limited central resources by incorporating a contingent capture task as the second task in a dual-task paradigm using N2pc as a marker of spatial attention. N2pc is an ERP component that appears in the right hemisphere if the participant pays attention to the left visual field, and vice versa, appears in the left hemisphere if attention is focused on the right visual field. The N2pc was significantly reduced in high concurrent central load conditions, indicating that even though it is involuntary, the deployment of visual-spatial attention occurring during contingent capture depends on capacity-limited central resources.

Soto and Humphreys (2008) found that when the WM item that was used as a cue for one of the distracters, did not match the subsequent search display, search performance was worse as compared to a neutral baseline. This effect of WM content on search was reduced when cognitive load was increased, and when articulatory suppression was used. Soto et al. (2008) reviewed evidence emanating from their lab regarding the influence of WM on search for relevant information from the environment. They contend that WM automatically guides selection, even if it is detrimental to performance. Further, on the basis of fMRI evidence (Soto et al., 2007) they assert that this modulation is a top-down process quite distinct from bottom-up processes such as priming. When a stimulus held in WM appeared in the search array, there was enhanced activity in the superior frontal gyrus, mid-temporal, and occipital areas. In contrast, implicit repetition priming (which involves mere repetition of a stimulus) elicited a suppressive response. Also WM probably affects the early process of attention that controls the entry of information into awareness. Soto and Humphreys (2009) assessed whether guidance by WM is limited to single task relevant dimensions, or does it differentially affect bindings of those dimensions. Participants were asked to remember the shape of a colored object in memory. Then they were to search for a target line amongst distracter lines, each embedded within a different object. On some trials, one of the distracter objects in the search display matched the memory item on the shape, the color, or both dimensions. Relative to a neutral baseline, where there was no match between the memory and the search displays, search performance was reduced when a distracter object matched both the color and the shape of the memory cue, demonstrating that WM had a greater impact on bindings as compared to single dimensions. Relevance of stimulus input to task goals thus seems to be the overriding factor in the process of binding.

## What happens to irrelevant input? discarded at the outset or gradually inhibited?

Searching for direct evidence, we (Jaswal and Logie, 2011; Logie et al., 2011) studied the effect of task relevance of features in the process of feature binding at study-test intervals from 0 to 2500 ms. All experiments used a version of the change detection task. The task presents two stimulus arrays to the participant who has to decide whether there is a change between the two arrays. The task requires not only the formation of mental representations, but also the maintenance or storage of these representations across time so that they can be compared in successive frames. As such, it is a perceptual as well as a memory task. Simply by manipulating the study-test interval one changes it from a test of perception to a test of memory.

The difference in the change detection task, if it occurs, may be in terms of the addition of a new stimulus, deletion of an old one, or a swap in the already presented stimuli. Binding is required only for the last kind of change, a swap between two stimuli. In fact, the swap detection task was introduced by Wheeler and Treisman (2002) specifically to study bindings. It is not possible to perform this task by remembering which features were presented, for all the same features appear in the study as well as the test display. It is essential to remember how the features were “combined” to find which ones swapped. Alvarez and Thompson (2009) have used the term “feature switch detection” to describe this task. Their work has also shown that although this task underestimates the binding capacity of VWM, it is an efficient paradigm for studying the factors affecting the fragile nature of bindings.

Since the aim was to study the effect of an irrelevant feature in an experiment, reducing the binding problem to its essentials, stimuli were defined concomitantly by three properties. The three features chosen to define the stimuli were location, shape, and color. The operational problem was how to design a task that would “break off” one of these elements to study the link or “binding” between the other two. One solution could have been to hold this element constant. For example, presenting various shapes in various locations, and swapping any two, whilst keeping the color of all the stimuli unchanged. Indeed this has been the procedure followed by many researchers in the field (e.g., Vogel et al., 2001; Wheeler and Treisman, 2002). However, it is questionable how far this manipulation prevents the inclusion of the irrelevant feature in the bound representation on each trial. If a feature is present constantly, it can still function as an informative cue. In fact, other features may be accessed through this feature. On the other hand, it may also block the effect of other features.

In the literature on conditioning, following Rescorla (1967), it is well established that the way to make a stimulus truly irrelevant and non-informative, is to randomize it. This idea was applied to the design of experiments by randomizing one feature between the study and test displays to render it non-informative, while testing memory for the combinations of the remaining two features of each object in the array. Using the swap detection task, location was randomized between the study and the test display with memory tested for the color-shape combinations in the study display. Analogously, shape was randomized to study the link between location and color, and color was randomized to study the link between shape and location.

In each case, binding between two of the features was studied whilst the third was rendered irrelevant through randomization. The focus was to study how far performance would be disrupted when a feature was rendered irrelevant through randomization in comparison to a condition in which it was unchanged. If there were no differences between unchanged and randomized conditions, it would indicate that participants can remove the unwanted irrelevant features right from the outset in accordance with task instructions. Reduced performance in the randomized feature condition would suggest that all features automatically participate in the initial representation even if they are irrelevant to the task. If a convergence occurs over time, it would suggest that relevant features can be consolidated and irrelevant features can be inhibited only gradually through the control processes of VWM. Thus, performance in the randomized feature condition was compared to when the feature was unchanged, to study *whether and when* the feature could be deleted from the visual system.

It was expected that performance in detecting change in bindings would be reduced when a feature is randomized from study to test as compared to when it is unchanged, if the feature had an initial representation, despite that the instructions were to ignore the feature and it was rendered completely non-informative and irrelevant to performance. It was also expected that as the visual system consolidated the binding of relevant features, this irrelevant feature would be inhibited, leading to a convergence of performance at longer study-test intervals.

This expectation was confirmed across experiments where locations were the irrelevant feature and color-shape bindings were to be detected. There was a convergence of performance at study-test intervals of 1500 ms or beyond. A similar pattern of interaction was also found when shapes were randomized to study color-location binding, and when colors were randomized to study shape-location bindings (see Logie et al., 2011, for details). This not only suggests that the effect is very robust, but also attests to the overriding importance of top-down factors in binding irrespective of the features involved. All features are treated the same way in VWM. They are selectively consolidated if they are relevant, and removed from the mental representation if they are irrelevant.

In the randomized condition, the task used in these experiments is a further variant of the swap detection task in the sense that in the test display, not only does the target change, but the distracters also change. The task becomes even more difficult, for participants have to decide whether there is a change in the binding of two features, when the third feature also changes. They have to ignore the changes in the one feature, and focus on finding the swap in the other two. This presumably involves a more demanding and central cognitive process in which the subject has to consider each of the stimuli in the test array, and compare whether the binding is the same as for the ones he holds in his memory.

Nonetheless, there is no denying the differential processing of features. In line with physiological studies (Zeki et al., 1991; Moutoussis and Zeki, 1997; Lamberts, 2002, Aymoz and Viviani, 2004) and psychophysical evidence (Magnussen et al., 1996; Magnussen and Greenlee, 1997; Magnussen, 2000), differential processing of features was found. There is greater disruption of performance when locations are randomized than when shapes or colors are randomized, with the disruption due to randomization of colors being the least. In addition, the removal of locations from the initial representation takes a much longer time than the removal of shapes or colors.

The differences among the three features, locations, shapes, and colors, follow the differential perceptual processing of these features. Location swaps are easiest to detect and location is the most difficult feature to ignore. Color swaps are the most difficult to detect, and color is the easiest feature to ignore. The results for shape fall between these two. This is in consonance with researchers showing that locations are processed in the dorsal stream, which is relatively automatic and works on an earlier time scale than the ventral stream (Velichkovsky, 1982, 2007; Vecera and Palmer, 2006). Between shape and color, differentiation of forms happens before the surface features are filled in (Cinel and Humphreys, 2006; Humphreys et al. 2000, 2009). To be speculative, the differential processing of features might happen with other features such as orientation, size, textures, etc., as well.

The differences in the amount of disruption experienced by the participants, imitate the importance of the “to be ignored” feature in our daily lives. The disruptive effect is least when color is the feature to be ignored, with a greater amount of disruption when shape is to be ignored, and the maximum disruption when location is to be ignored. The correct perception of the location of objects in space has survival value in our daily navigation of the world, and reflecting that importance, randomizing location disrupts performance to the greatest extent in these experiments.

These results go against the overwhelming importance accorded to locations by many researchers (e.g., Wolfe, 1994; Treisman, 2006). Perception may be location based, but memory may be not only location based, it might well be object based, or feature based. Just as it is possible to ignore other features, it is possible to ignore locations too. It is only more difficult, not impossible. As compared to other features, location is special. But, in itself, it loses its importance in the binding process if it is not relevant. Thus relevance of features overrides the differential processing of features.

### Summary so far

The account of the binding process that emerges is that features may not be bound together instantaneously and all at the same time. Instead, their processing in the visual system continues at different rates. This differential processing affects *when* they are bound in object representations. Object representations involving shape-location bindings are formed most easily or are the strongest, followed by color-location bindings, followed by shape-color bindings. Certainly, there is no clear, coherent, strong object right from the outset. It is also clear that there is a selective process that binds only some features together. The task relevance of features determines *whether or not* they are bound into the object representation, i.e., they are bound in the object weighted by their task relevance. Features are consolidated if relevant, and discarded if irrelevant, but only as a gradual process.

## Is inhibition a perceptual or post – perceptual process?

Any discussion regarding binding of relevant features cannot be complete without discussing “relevant for what?” Adaptive organisms that we are, the ultimate goal of the process of binding is necessarily some sort of action. Kahneman et al. (1992) suggested that we create object files that contain all the perceptual information about an object. Nevertheless, as discussed, binding is not restricted to perception. Via WM, it is linked to actions. As such, the ideas of “instances” (Logan, 1988) or “event files” (Hommel, 1998, 2004, 2005) containing perceptual as well as action related information assume importance. Indeed, Davelaar (2011) postulates that all real world tasks can be denoted as “goal representations” which are bindings of their sub representations. The sub representations are for perceptual inputs, conceptual rules, and/or motor responses bound together by a memory trace. One infers that the sub representations are themselves bindings as well, for example, comprising the task relevant features of sub-states, or the properties of task-specific control representations.

Hommel (2004) postulated the importance of task relevance as a factor at the time of initial binding. However, experimental evidence shows that the inhibition of irrelevant features is gradual and requires some time to occur (Logie et al., 2011). In another study (Jaswal and Logie, 2011), the display duration was increased from 200 to 900 and 1500 ms. This improved performance overall, but had no differential effect at the two study-test intervals of 0 and 2000 ms. This indicated that the inhibition of irrelevant features did not happen during the presentation of the study display, and is not operational during encoding, but that it is a post-perceptual process within WM (see Experiment 1, Jaswal and Logie, 2011, for details). Inhibition was used in many different ways in this experiment. The stimuli in the study display always being above capacity, focusing is required to select a region, objects, or features. This uses the prioritization function of attention. This selection process necessitates that the rest of the locations, objects, and features are deselected. These would not influence or reappear in performance. Thereafter, from the selected representations, of features, objects, or locations, there is a process of removal of the irrelevant, unwanted feature. This process begins only after encoding is complete. This crucial process is presumably a part of the central executive component of WM, and comes into play to extract meaning from an otherwise confusing array of stimulus dimensions. Gradually, this inhibitory process is complete, and the object comprising relevant features emerges to be maintained in WM, ready for further processing. Supportive evidence comes from fMRI studies by Sala and Courtney (2007) who found reduced activity over time in reaction to “conjunction” stimuli in cortical regions dedicated to “what” and “where” stimuli. Interestingly, this reduction does not happen for only “what” or only “where” stimuli. It happens only following bound stimuli which use both these streams of processing. Analogously, a number of studies with the preview search procedure (which is very similar to the task described here) have suggested that “active inhibition” is a higher order process that follows the initial registration of the stimuli (reviewed by Soto et al., 2008). Thus, inhibition of irrelevant features occurs over time and is presumably a process within WM.

This inhibitory process is rather different from the orientation function of attention that allows selection of locations and/or objects from the stimulus display that is presented. Indeed it is possible to focus and use this latter type of attention even before the stimuli are displayed or in the complete absence of distracters (Henderson, 1996). In contrast, the inhibitory process occurs after stimulus presentation and seems to be directed at everything that is irrelevant in the stimulus display – be it features or objects. In this sense, it is similar to distracter suppression, which appears only after the distracters are identified (Luck and Hillyard, 1994a,b; Luck et al., 1997; Luck, 1998).

In the area of WM, an inhibitory process was first proposed by Hasher and Zacks (1988) to account for differences among older and younger adults in WM. They proposed that successful processing implied allowing relevant information in and keeping irrelevant information out. However, they did not apply this idea to features within bindings, and restricted their view to objects in WM. Subsequent studies have shown that the memory problems of older adults are not so much regarding individual features but about bindings of those features (Chalfonte and Johnson, 1996; but, see Brockmole et al., 2008). The gradual process of deleting or inhibiting a feature that is task-irrelevant and possibly disruptive has been identified as an important aspect of WM executive functions (Miyake et al., 2000; Friedman and Miyake, 2004).

### Summary so far

All features and objects, indeed the whole display, enter the sensory register of the participants, and gradually, from this representation, the relevant features and objects are selected and retained, and the irrelevant ones are discarded. Whether the features participate in a preliminarily integrated percept to be refined thereafter or whether the features are held in separate feature maps is a moot point. The vast literature on information processing theory has shown that parallel representation of stimulation followed by serial decision making is a much more efficient procedure, than selecting each object one by one and making decisions about it (e.g., Sternberg, 1966, 1967). It being easier to encode all stimuli and then make the decisions, participants might loosely represent the irrelevant as well as relevant features initially, deleting the irrelevant ones only after the display is gone, and no more relevant features can be committed to memory. Certainly, there is no clear, coherent, strong object right from the outset. The deletion of features from within a representation, such that it becomes a coherent strong object capable of further manipulation, takes time and resources, and is a preliminary phase in the online processing of objects in WM. Thus, the emergence of the bound object is a result of an active inhibitory process in WM.

## Conclusion: The process of binding

**Figure 1:**
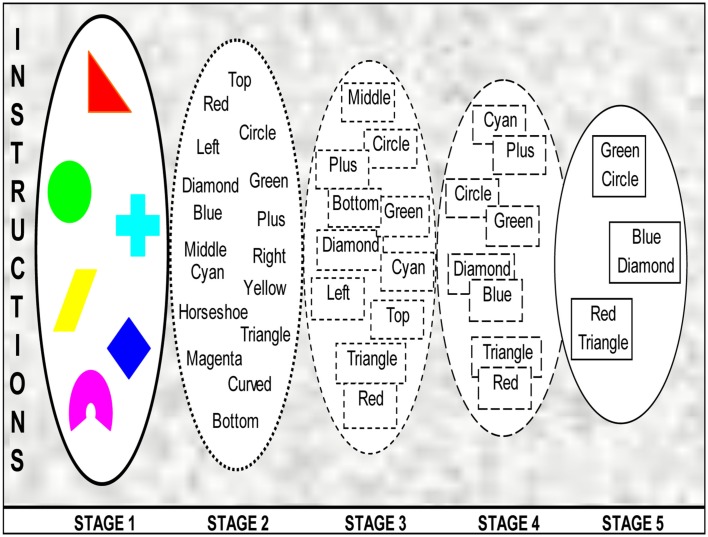
**The process of binding**.

The process of binding as understood on the basis of research reviewed above is illustrated in Figure 1. The five stages represent cross-sections of the process to aid understanding, otherwise the process is assumed to be continuous. The area covered by the ovals gradually reduces to depict the decrease in the amount of information available to the participant, and also increasingly focused attention. It is, nevertheless, accepted that attention plays different kinds of roles in this process. The gradual completion of the boundaries is used to show the increasing clarity of the representations.

The whole process is dictated and delimited by instructions from WM which define the goal for the participant. Even before the trials begin, the participant is set to ignore the irrelevant feature. Stage 1 represents stimuli in the real world. Stage 2 is the initial representation of the stimulus dimensions, which includes almost all the various features defining the stimuli. However, there is some loss of information even at this early stage, as a proportion of the stimuli impinging on the sense organs are selected to be processed further. This stage contributes to the binding process by holding information as an almost veridical representation of reality which can be organized and further consolidated. It corresponds to retinotopic iconic memory, and is vulnerable to an immediate mask. It is also difficult to build up this representation with sequential presentation when one item vanishes as the subsequent one is presented.

Stage 3 is a spatiotopic representation with a greater loss of information than Stage 2, but which has much more information available than the later stages. Presumably it corresponds to the fragile VSTM proposed by Sligte et al. (2008). The process of active inhibition is dominant between the Stages 3 and 4, and removes the irrelevant feature, in this case locations. The inhibitory process is otherwise a necessary component of top-down processes which select relevant information and inhibit irrelevant information and operates throughout the binding process. This top-down processing is achieved by reentrant processes. Notice how reentrant processes recover the relevant feature “blue” to be amalgamated with “diamond” as represented in the next stage. Stage 4 represents only the relevant features, with increasing overlap between them to show the strength of binding at this stage. Stage 5 shows features bound as objects in VWM ready for further processing.

Though the illustration uses location as the feature to be ignored, it is assumed that analogous processes operate if color or shapes (or other stimulus dimensions) are to be ignored. The total duration of this process will vary with the stimulus dimensions involved. As per experimental evidence (Logie et al., 2011), the duration of the process is shorter when shapes or colors are to be ignored.

Can any information be directly transferred to WM at all? Is it possible for some information to bypass these stages and appear in VWM? One may speculate that the stage sequence is invariant, although the time scale can be considerably shortened if the broad attentional window includes a narrowly focused mechanism that achieves this. This narrow focus may be due to top-down factors, say, an “intention” to remember all red items, or all curved items, or the first item presented, or a red plus because it evokes associations with the Red Cross. Such an intentional focus would necessarily involve activated representations in LTM. Alternatively, it may at times, result from the higher activation level of a particular item due to bottom-up stimulus factors such as salience, first or last serial position, etc. It is the transactions between top-down and bottom-up processes which determine the course of the binding process, although the final outcome is contingent on the dictates of the task goals held in WM and the relevance of features to them.

## Conflict of Interest Statement

The author declares that the research was conducted in the absence of any commercial or financial relationships that could be construed as a potential conflict of interest.
